# Iron, Inflammation, and Malaria in the Pregnant Woman and Her Child: Saving Lives, Saving Brains

**DOI:** 10.4269/ajtmh.16-0533

**Published:** 2016-10-05

**Authors:** Sarah E. Cusick, Chandy C. John

**Affiliations:** ^1^Department of Pediatrics, University of Minnesota, Minneapolis, Minnesota; ^2^Department of Pediatrics, Indiana University, Indianapolis, Indiana

The battle for iron between host and pathogen in children living in malaria-endemic areas is fierce, and new evidence presented by Brickley and others^1^ in this month's issue suggests it may well begin before birth. In previous work, this group showed that iron deficiency in children living in a malaria-endemic area of Tanzania was associated with a decreased risk of malaria and death.^2^ In the present study conducted in the same cohort, the authors show that higher cord blood concentrations of the iron regulatory and inflammatory protein hepcidin predict a significantly greater risk ofanemia but a lower risk of malaria and all-cause mortality during early childhood. Maternal inflammation, placental malaria, and higher maternal hemoglobin concentration were all significantly associated with greater cord blood hepcidin. This high-quality study, with its large sample size, prospective design, and remarkable follow-up, is significant because it links iron and malaria dynamics in pregnancy with those of early childhood, specifically demonstrating that in malaria-endemic areas, susceptibility to childhood anemia, malaria, and even death may to some extent be determined before birth.

Hepatic production of hepcidin is known to have multiple drivers, including dietary iron sufficiency, inflammation, and slowed erythropoietic rate.^3^–^5^ The study by Brickley and others^1^ shows that in malaria-endemic areas, inflammation inparticular has an outsize influence on cord blood hepcidin concentrations, as conditions associated with inflammation (placental malaria, high malaria transmission season) and markers of inflammation (higher placental, maternal, and cord blood concentrations of inflammatory cytokines and C-reactive protein [CRP]) together predicted the greatest portion of variability in cord blood hepcidin concentrations. The study findings also suggest that in malaria-exposed pregnant mothers, limited maternal systemic iron availability—and perhaps consequent limited iron transfer to the fetus—may be protective for their children in terms of later malarial morbidity. In fact, such limited iron availability and transfer may set the stage for the naturally occurring iron deficiency in this infant cohort that the authors previously showed was associated with protection from malaria.

The question arises as to whether higher cord blood hepcidin, seemingly reflecting maternal inflammation and predictive of future childhood anemia but protection from subsequent malaria, is also a marker of chronic early childhood functional iron deficiency. Serial measurements of hepcidin, ferritin, and CRP throughout early childhood could confirm such a mechanism. In a birth cohort, this functional iron deficiency would manifest as high concentrations of hepcidin, ferritin, and CRP concurrent with a low hemoglobin concentration, and would reflect a state in which body iron was in reticuloendothelial stores and unavailable to invading pathogens, but also unavailable for red blood cells and the developing brain. Giving iron supplements in such a setting may at best be futile, as high hepcidin concentrations would limit dietary iron absorption,^6^,^7^ and could at worst be dangerous.^8^ This study thus provides further evidence to justify the current caution in the implementation of early childhood iron supplementation in malaria-endemic areas.

The study by Brickley and others^1^ shows that placental malaria is associated with increased cord blood hepcidin concentrations in neonates, which are in turn associated with a lower risk of malaria and death in early childhood. Yet, we know placental malaria is not good for the fetus: it is associated with prematurity and intrauterine growth restriction,^9^ and placental inflammation in other conditions is associated with impaired child neurodevelopment.^10^ Similarly, the current lack of iron supplementation programs for children in malaria endemic areas may save lives and protect children from malaria and other infections, but at a cost to the developing brain in survivors. Multiple processes, for example, myelination, and structures, for example, hippocampus and prefrontal cortex, of the brain that require iron undergo their peak rates of development during the last trimester of pregnancy and throughout the 1st year of life.^11^ One cannot argue with the withholding of iron during this time whether it reduces malaria and saves lives, but the lives may be saved at the tremendous long-term cost of injury to the developing brain.

The findings from the study by Brickley and others and the identification of high cord blood hepcidin as a promising biomarker of future risk of childhood infection and mortality raise important questions for future research and programmatic interventions concerning iron in malaria-endemic areas. The results underscore the necessity of effective malaria-control efforts through all stages of pregnancy and childhood as a prerequisite for safe attainment of sufficient iron status. However, they also raise the question of the relative contributions of dietary iron deficiency versus maldistribution of body iron to childhood iron deficiency in malaria-endemic areas. Could alleviation of inflammation and resultant release of iron from reticuloendothelial stores be sufficient for erythropoiesis and brain development, while obviating the need for iron supplementation? Would the resulting “iron sufficiency” still place the infants at higher risk for malaria and death in early childhood? Conversely, should children with low cord blood hepcidin concentration be put on intermittent preventive treatment of malaria and then be provided iron supplementation? These questions need to be answered in well-designed randomized clinical trials with both short-term morbidity and long-term neurodevelopmental outcomes. Assessment of the latter outcomes will help to answer the additional question of the extent to which iron supplementation in infants in malaria-endemic areas improves neurodevelopment, and which forms of iron supplementation most effectively improve child neurodevelopment. Although it is unlikely that iron deficiency is good for a child's developing brain in any part of the world, the effectiveness of iron supplementationto prevent or reduce child neurodevelopmental impairment in malaria-endemic areas has not been studied, largely because of concerns about the general safety of iron supplementation.

The study by Brickley and others sheds an important and clarifying light on links between iron and malaria dynamics in pregnancy and early childhood, and identifies cord blood hepcidin as a promising biomarker for prediction of key child health outcomes in children born to mothers with malaria-induced and possibly other infection-induced inflammation. If confirmed by other malaria-in-pregnancy studies, these findings set the stage for future pathogenesis studies and clinical trials that aim to save children's lives while preserving their developmental potential.
